# Tandem repeats lead to sequence assembly errors and impose multi-level challenges for genome and protein databases

**DOI:** 10.1093/nar/gkz841

**Published:** 2019-10-04

**Authors:** Ole K Tørresen, Bastiaan Star, Pablo Mier, Miguel A Andrade-Navarro, Alex Bateman, Patryk Jarnot, Aleksandra Gruca, Marcin Grynberg, Andrey V Kajava, Vasilis J Promponas, Maria Anisimova, Kjetill S Jakobsen, Dirk Linke

**Affiliations:** 1 Centre for Ecological and Evolutionary Synthesis, Department of Biosciences, University of Oslo, NO-0316 Oslo, Norway; 2 Faculty of Biology, Johannes Gutenberg University Mainz, Hans-Dieter-Husch-Weg 15, 55128 Mainz, Germany; 3 European Molecular Biology Laboratory, European Bioinformatics Institute (EMBL-EBI), Wellcome Genome Campus, Hinxton. CB10 1SD, UK; 4 Institute of Informatics, Silesian University of Technology, Akademicka 16, 44-100 Gliwice, Poland; 5 Institute of Biochemistry and Biophysics PAS, Pawińskiego 5A, 02-106 Warsaw, Poland; 6 Centre de Recherche en Biologie cellulaire de Montpellier, UMR 5237 CNRS, Universite Montpellier 1919 Route de Mende, CEDEX 5, 34293 Montpellier, France; 7 Institut de Biologie Computationnelle, 34095 Montpellier, France; 8 Bioinformatics Research Laboratory, Department of Biological Sciences, University of Cyprus, PO Box 20537, CY 1678 Nicosia, Cyprus; 9 Institute of Applied Simulations, School of Life Sciences and Facility Management, Zurich University of Applied Sciences (ZHAW), Wädenswil, Switzerland; 10 Swiss Institute of Bioinformatics (SIB), Lausanne, Switzerland; 11 Section for Genetics and Evolutionary Biology, Department of Biosciences, University of Oslo, NO-0316 Oslo, Norway

## Abstract

The widespread occurrence of repetitive stretches of DNA in genomes of organisms across the tree of life imposes fundamental challenges for sequencing, genome assembly, and automated annotation of genes and proteins. This multi-level problem can lead to errors in genome and protein databases that are often not recognized or acknowledged. As a consequence, end users working with sequences with repetitive regions are faced with ‘ready-to-use’ deposited data whose trustworthiness is difficult to determine, let alone to quantify. Here, we provide a review of the problems associated with tandem repeat sequences that originate from different stages during the sequencing-assembly-annotation-deposition workflow, and that may proliferate in public database repositories affecting all downstream analyses. As a case study, we provide examples of the Atlantic cod genome, whose sequencing and assembly were hindered by a particularly high prevalence of tandem repeats. We complement this case study with examples from other species, where mis-annotations and sequencing errors have propagated into protein databases. With this review, we aim to raise the awareness level within the community of database users, and alert scientists working in the underlying workflow of database creation that the data they omit or improperly assemble may well contain important biological information valuable to others.

## INTRODUCTION

The availability of DNA and protein sequence data has revolutionized the way we study cellular, molecular, physiological, evolutionary and developmental processes, allowing the association of phenotypes with genotypes at a single nucleotide (or single amino acid) resolution. Researchers rely on public sequence depositories and other databases for sharing their data, such as GenBank or UniProt, and the content of these databases has grown exponentially in the last decades. While such databases initially consisted predominantly of submissions of individual gene or protein sequences that were carefully curated, large proportions of the content of genome and protein databases today originate from different types of metagenome and genome sequencing and assembly projects. GenBank, for example, included more than 2635 Gbp (billion base pairs) in its 2017 release number 221, of which 2242 Gbp (85%) originated from whole-genome shotgun sequencing (1). For an informed use of such data, it is essential that end users understand the distinct contrast in quality between individual, well-curated submissions and entries generated from automated sequence annotation pipelines. The latter procedures can contain unrecognized errors.

Here, we argue that awareness of potential database errors is especially relevant with regards to repetitive stretches of DNA, which can occur in both noncoding and coding regions of genomes. The specific nature of this type of DNA sequences can introduce and propagate bias during multiple levels of analyses, and resulting uncertainties and errors are automatically translated further into protein sequences where they become impossible to recognize. Such issues may arise from problems originating from DNA sequencing, from difficulties with assembling repetitive DNA regions and from inaccuracies generated during the annotation process. The multiplicity of these error sources makes it particularly difficult for researchers to understand and assess the bias that may be underlying the sequences that they retrieve from public databases. As an example, in Table 1, we have listed the total number of proteins in UniProtKB/Swiss-Prot that have changed the length of their repetitive region from the first occurrence in the database to the latest—suggesting that errors in repetitive region length have been identified and corrected. The average difference in length is 13.57 amino acids, a substantial number. The 1669 proteins with differences in repeats (Table 1) are 6% of all proteins in the database that have a repetitive region (see Table 2). These numbers do not reflect a true error rate but suggest that errors in repeat numbers and repeat length are frequent and might often go unnoticed, especially in databases that are less well curated than UniProtKB/Swiss-Prot.

**Table 1.** Summary of proteins from UniProtKB/Swiss-Prot where the length of repetitive region has changed between different versions of the database

Proteins (*n*)	Proteins with different sequence between versions (*n*)	Proteins with different repetitive region lengths (*n*)	Average/standard deviation of the length of repetitive regions in original version of the sequence^a^	Average/standard deviation of the length of repetitive regions in the version 2018_06 of the sequence^a^	Average/standard deviation of the difference in lengths of repetitive regions^a^
554 241	74434	1669	31.14/72.09	35.20/84.08	13.57/45.69

^a^Measured in amino acid residues.

**Table 2.** Differences of repetitive region lengths in evolutionarily distinct groups of organisms

Database name	Number of proteins	Number of proteins with STRs	% of proteins with STRs	Median^a^	Average^a^	Standard deviation^a^	Number of clusters^b^
UniProtKB/Swiss-Prot (total)	554 241	28003	5.05%	14.75	15.14	3.69	6237
Archaea	19 525	351	1.80%	10.71	10.63	1.27	45
Bacteria	333 691	6794	2.04%	17.38	17.45	2.66	1048
Euk: Fungi	33 613	3996	11.89%	13.46	13.79	3.65	893
Euk: Invertebrata	27 607	3372	12.21%	17.34	18.62	7.95	812
Euk: Vertebrata	18 292	1461	7.99%	13.66	13.90	2.42	1801
Euk: Plants	42 101	3601	8.55%	12.51	12.82	2.98	795
Viruses	16 852	889	5.28%	14.07	14.15	2.57	203

^a^Repetitive region length, measured in amino acid residues.

^b^Clustering was used to define repeat classes. Should a protein contain three different, co-localized STRs, the clustering method will produce 6 clusters: three with regular STRs and three with fused repeats. See also supplementary material for more information.

In this review, we discuss different types of sequencing and database errors, using prominent, published examples where such errors have been found. We first provide a description of the different types of repeats that occur on the DNA and protein level and an overview of DNA sequencing technologies with their benefits and limitations. We then describe the genome assembly, annotation, and database deposition processes, and then link these processes to the different types of errors that may occur at different points in this workflow. We aim to alert the ever-growing community of database end-users of these errors, and to raise awareness among the scientists working in the underlying workflow of database creation, that data that they omit or improperly assemble may well contain important biological information valuable to others.

### Repetitive elements in genomes

Repetitive DNA occurs in all domains of life—Bacteria, Archaea and Eukaryota—and can be grouped into two categories: interspersed repeats, such as transposable elements occurring in multiple loci across the genome, and tandem repeats (TRs) that occur in a single locus. In eukaryotes, repetitive DNA also occurs in specific chromosomal regions, such as the (sub)telomeric regions (2,3) and the centromeres (4). Transposable elements (TEs) are typically several thousand base pairs (kbp) in size, and in eukaryotes their size can range from 100 base pairs (bp) to 20 kbp (5). Large fractions of vertebrate genomes are filled with active and inactive fragments of TEs, with more than 40% of the genome of zebrafish and more than a third of mammalian genomes consisting of TEs (6). Evolutionarily old TEs will accumulate mutations and will diverge from the original sequence, and TEs can therefore lose their repetitive nature over time. In contrast, TRs may consist of motifs as short as 1 bp, where the motif is repeated in tandem. Short tandem repeats (with a motif shorter than 10 bp) were originally called microsatellites (7), longer tandem repeats (with a motif between 10 and 100 bp) were called minisatellite DNA (8), and long tandem repeats (with a repeating motif longer than 100 bp) were called satellite DNA (9). In eukaryotes (based on studies done on metazoans, green algae, plants and yeast), the content of TRs with a unit size of 1–50 bp usually varies between 2000 bp/Mbp and 55 000 bp/Mbp (corresponding to 0.2–5.5% of the genome) (10,11). Repeats also lead to significant intra-specific variation (i.e. variation between individuals of the same species) (12,13) as shown in a wide range of eukaryotes, for instance *Arabidopsis* (13,14) and *Drosophila* (15). Within humans, repeats outnumber the number of bases affected by SNP variation by an order of magnitude (4–5 fold) (16). Intra-specific variation poses its own intrinsic challenges for instance when sequencing samples from pooled individuals (17). Short tandem repeats (STR) are less prevalent in bacteria compared to eukaryotes—presumably due to the typically compact bacterial genomes—but nonetheless regularly occur in bacterial coding regions (18).

TEs can cause ‘breakage’ of a continuous assembly and lead to assembly collapse, where the number of copies of a repeat found in a genome assembly is lower than the true number, but the relatively large and often evolutionary divergent TEs are unlikely to greatly affect the accuracy of sequencing, assembly and annotation of individual protein-coding regions. While such TEs might sometimes insert themselves into gene regions, the disruptive effects of multiple kbps of sequence inserted into coding regions likely make these events extremely rare. In contrast, TRs are usually much shorter, and can often be in-frame in coding regions; therefore, we mainly focus on the problems caused by this class of repeats on the sequencing, assembly, annotation and database deposition processes.

### Short and long tandem repeats in coding sequences

TRs are found in both non-coding and coding genomic regions, and the latter make repeated sequences also ubiquitous in proteomes. Conservative estimates suggest that TRs are present in at least one third of human protein sequences and in half of the protein sequences of the unicellular malaria parasite *Plasmodium falciparum* and the mold *Dictyostelium discoideum* (19,20). In UniProtKB/Swiss-Prot, 5% of all proteins have a repetitive region (see Supplementary Material and Table 2). The TR regions come in various flavors; from single amino acid repeats (homorepeats) to the repetition of homologous domains of 100 or more residues (21,22). TRs with short repetitive units are more frequent than those with long repetitive units (19,23,24), and repeats are more frequent in Eukaryota compared to Bacteria and Archaea (Table 2). With their highly mutable nature, the presence of variable TRs in coding sequences may directly lead to an increase in protein variation and modification, which is particularly relevant for functional and evolutionary studies (25,26).

Tri-nucleotide repeats in coding regions may result in amino acid homorepeats (or polyX). These are widely distributed in all branches of the tree of life and in many protein types (27). Like other TRs, homorepeats can be important for function and their length variation is modulated by selection, as has been demonstrated for many protein families (28). In particular, the expansion of CAG repeats that translate to polyglutamine tracts (polyQ) have been widely studied. These polyQ stretches seem to be advantageous for function in protein interactions. When the length of the repeats is too long, the resulting proteins can aggregate and cause disease, leading to selection against further repeat expansion (29). Dedicated databases and resources have been developed to list and characterize amino acid homorepeats of all types (30,31).

Approximately half of the TR regions in proteins may be naturally unfolded (32–34), while the other half of these repetitive regions folds with a plethora of shapes and functions (35,36). Their protein structures can be subdivided into five major classes: (i) crystalline aggregates formed by regions with 1 or 2 residue long repeats, (ii) fibrous structures stabilized by interchain interactions with 3–7 residue repeats, (iii) structures with the repeats of 5–40 residues dominated by solenoid proteins, (iv) ‘closed’ (not elongated) structures with 30–60 residue long repeats and, finally, (v) ‘beads on a string’ structures with typical size of repeats over 50 residues, which are already large enough to fold independently into stable domains (35,36). When studying repetitive protein structures, it is essential that the underlying sequence information is accurate, not only regarding the type of repeats, but also the exact repeat unit number, as the latter will for example influence the length of protein fibres or the curvature of solenoid proteins. Unexpectedly high conservation of TR repeat unit number and order has been reported for proteins from species separated by long evolutionary time (23,37). This implies that negative selective pressures act on TRs to preserve important protein functions. The same studies suggest that diversifying selective pressures may play equally important role in function of TR-containing proteins. For example, leucine-rich repeats can be both conserved and play role in adaptation (37–39). Indeed, consistent with this premise, TRs are frequently found in virulence factors of pathogens, toxins, allergens, amyloidogenic proteins and other disease-related sequences. Fast-evolving repeat regions might confer variation to the surface proteins of pathogens allowing them to escape the host defense systems (40,41). Moreover, there is an increasing amount of evidence for a causal relationship between mutations in TR regions and human-inherited genetic disorders (42). All these examples show that errors in databases are not only an academic problem but also pose risks in analyses of medically relevant data.

In the following sections, we discuss different problems that occur in today's sequence databases. All these problems originate directly or indirectly from the sequencing and assembly process, and all relate to repeats on the DNA level, leading to fundamental errors in the final database entries.

## SEQUENCING AND GENOME ASSEMBLY ARE AFFECTED BY TANDEM REPEATS

### High-throughput sequencing technologies

High-throughput sequencing technologies remain under fast development and several types of technology have been or are currently available. Each of these technologies has its own distinct features that influence their ability to characterize repeats. In the Sanger sequencing technology era, each read was accompanied by a fluorescent peak trace chromatogram. This enabled researches to double-check whether or not the correct base was incorporated in a position, which could be helpful in troublesome regions such as repeats. While similar information is available for high-throughput sequencing technologies, usually encoded as quality scores, the massive amounts of data produced makes it infeasible to manually check the quality of individual bases.

The most widely-used technology is the Illumina sequencing platform (43). This technology has a relatively low sequencing error rate (<0.1%) (44), and errors are mainly due to substitution errors. Nonetheless, Illumina reads are relatively short (<250 bp), which is a limiting factor since many repeat regions are longer than the length of the read. This technology is therefore not able to fully resolve such longer repeats.

Platforms with significantly longer read length comprise the Single Molecule Real Time Sequencing from Pacific Biosystems (‘PacBio’) (45) and Nanopore Sequencing from Oxford Nanopore Technologies (‘Nanopore’) (46). The longer read lengths (1–100+ kbp, usually 10–40 kbp) can successfully span longer stretches of repetitive DNA such as TRs and TEs. Both platforms, however, have high single-pass error-rates (11–15% for PacBio (47), similar for Nanopore (48)). The majority of these errors consist of insertion and deletions (indels), leading to additional or fewer nucleotides compared to the actual genomic sequence. These error rates can be addressed by more sequencing data (to a higher coverage), which will allow for better error correction during assembly. This effort comes at considerable additional economic costs, which can be up to an order of magnitude more expensive than Illumina sequencing.

A discontinued platform is the Roche/454 pyrosequencing technology. Producing reads up to 1000 bp, the 454 technology had difficulty with accurately sequencing homopolymers, leading to indel errors in such regions (49). Albeit 454 finds nearly no use for whole-genome sequencing today, data obtained from this technology still constitutes a considerable part of the DNA and protein sequence databases, being the platform with the second most entries in SRA still today (see Supplementary Material). The Ion Torrent system is similar to the Roche/454, and also has similar issues with indels (50). The relatively long read lengths of these technologies have benefits for crossing repeat regions, yet this advantage is somewhat negated by their inability to correctly assess longer (>4–5 nucleotides) stretches of homopolymers (51).

It is clear from descriptions above that in a perfect world, all sequence data generated would consist of high-coverage, long-range PacBio or Nanopore sequencing as a basis, with some Illumina data for error correction. Yet, the short Illumina reads are economical, accurate and can resolve most parts of any genome, which includes most coding regions and degraded TEs. The economy and utility of the Illumina platform is the main reason why so many genomes have been and are still sequenced by that technology, even though PacBio and Nanopore sequencing would technically yield more complete genome assemblies. Given the widespread use of Illumina technology, genome assemblies and databases are currently likely biased against longer TRs in that many of them do not get incorporated into assembled sequences. How this impacts or biases protein databases cannot be quantified, but individual examples show that especially data from short-read technologies must be taken with care when working with repeat proteins; we show some of these examples in detail further below. We do know that large fractions of proteins in protein databases do contain short TR regions (5% in UniProtKB/Swiss-Prot, Table 2) and that some of these have had changes in their TR region length from one ‘version’ of the protein to another (Table 1). Taken together, it is likely that protein databases underrepresent TRs and that many of the TRs that are in these databases are not correct.

### Genome assembly methods

The process of genome assembly creates a tentative reconstruction of a complete genome based on information found in the sequencing reads and possibly other sources of information, such as linkage maps. There are two major approaches for genome assembly, the ‘*de Bruijn graph*’ and ‘*overlap/layout/consensus (OLC) methods*’ and these differ significantly in how repeats get resolved during the assembly process.

The *de Bruijn graph* method uses subsequences (*k*-mers) found in the reads and creates a graph where each node represents a fixed-length sequence (*k*-mer), and the edges connect two *k*-mers with *k* – 1 bp sequence in common (which can be found in multiple reads) (52). This graph is then parsed, and depending on implementation, contigs (contiguous sequence based on consensus sequence from the reads) and scaffolds (contigs ordered and oriented based on paired read information) are generated. For the *de Bruijn* approach, the length of an entire repeat region has to be shorter than the *k*-mer (which is usually between 21 and 96, with 31 often used as the default setting) to be properly resolved. For instance, the *de Bruijn graph*-based assembler ALLPATHS-LG collapses all repeats equal to or longer than 96 to 96, its *k*-mer size, in its first processing stages (53), but the repeats can be expanded later in the assembly process. Newer implementations of the de Bruijn approach, such as SPAdes (54) and SKESA (55), use multiple *k*-mers to better assemble low sequence coverage regions and repeats. However, neither are designed to assemble larger (such as plant or vertebrate) genomes.

One implementation of the *OLC method* was Celera Assembler, which was used to assemble the *Drosophila* genome in 2000 (56), the first whole genome shotgun sequencing project of a multicellular organism. This approach works by first detecting overlap between all sequencing reads, then creating a graph based on the overlaps, simplifying and traversing the graph, before outputting so-called unitigs (sequences that are either unique in the genome or are collapsed, repeated sequence where repeats occurring in multiple locations in a genome are all found on top of each other in one sequence), based on a multiple sequence alignment from the overlaps (57). Because the overlap step compares each read to all other reads, computational demand can be high (certainly higher than the *de Bruijn* method), but it is reduced with fewer but longer reads because fewer overlaps need to be computed. The overlap step can also tolerate mismatches and indels between the reads, and therefore performs well with longer reads even if these are error-prone. The unitigs are further categorized into unique and repeat unitigs, before they are ordered and oriented into scaffolds based on information from paired reads (if included in the assembly). The *OLC method* can resolve those repeats that are shorter than the read length, and it is not limited by any *k*-mer size as the *de Bruijn* method. Before the availability of long reads such as PacBio and Nanopore, the shorter Illumina reads were usually assembled with the *de Bruijn* method because *OLC* can be computationally demanding. Now, with long reads decreasing in cost, most genome sequencing projects utilize these and assemble them with an assembler implementing *OLC*. This will lead to more complete genomes being published, with more repeats resolved.

### Repeat content and fragmented assemblies

While the choice of best-practice sequencing methods and assembly approaches can be used to minimize the effects of repeats, their amount, length, localization and sequence identity constitute key limitations to obtaining a complete and contiguous genome assembly (58). TE content is likely the largest factor contributing to fragmented genome assemblies (59). This holds for both assemblies based on Illumina and for PacBio reads, but the problem is larger for assemblies with shorter reads. TE content is part of the reason why larger genomes are harder to assemble, since it is highly correlated with genome size (6,60). While TEs might induce gaps in the genome assembly, the effects of TRs are harder to quantify. It is not completely clear how PacBio reads handle long STR regions. In one study (61), the authors investigated how PacBio reads handled different STRs, and showed that <50% of reads called the correct length of a STR consisting of 30xAC, most likely due to polymerase slippage errors. This observation partly contradicts the notion that long reads might be the solution to resolving repetitive regions (see conclusions section). However, such slippage problems appear limited to extreme examples, and overall, PacBio-based assemblies using *OLC* should be more accurate than Illumina-based assemblies with regards to STRs (62).

## EXAMPLES OF REPEAT-DRIVEN ERROR PROLIFERATION

### Tandem repeats cause sequencing and genome assembly challenges

Significant variation in the natural abundance of TRs exists in different organisms which complicates assembly procedures and the development of adequate algorithms that perform well in all cases. Atlantic cod (*Gadus morhua*) has been identified as a vertebrate species with an exceptionally high occurrence of STRs (63,64), in particular AC dinucleotide repeats (62,65). The high abundance of these repeats has caused several complications, both from a laboratory and bioinformatic perspective, and on the level of DNA and (translated) protein sequences. The first *de novo* assembly (gadMor1) of the Atlantic cod genome was based on 454 sequencing data (66) and resulted in a fragmented assembly with many gaps. More than 30% of the contig edges contained an STR and nearly a quarter of the gaps in scaffolds were flanked by STRs (Supplementary Note 7 in (66)), indicating that these STRs strongly affected the successful assembly into more contiguous genomic regions. By incorporating PacBio reads, an updated assembly (gadMor2; (62)) yielded an improved continuity, allowing a more in-depth quantification of these repeats. For instance, the antifreeze glycoproteins were completely missing in the gadMor1 assembly (67), while they are found in gadMor2 (see section ‘*Tandem repeats can hinder proper gene annotation*’ below). While it is well established that repeats in general can hinder genome assembly, there is little discussions about TRs in particular in the literature besides the example above. For instance, in a discussion regarding fragmented genome assemblies of plants, the authors do discuss briefly the role of TEs in the fragmentation of the assemblies, but never mention TRs in the same setting (68). When discussing repeat content, they only mention TEs. They further mention long reads as the main aid in generating more complete genome assemblies.

The prolific STR occurrence in Atlantic cod may also interfere with PCR amplification, often an essential step for creating sequencing libraries. Ancient DNA (aDNA) sequencing data from historic Atlantic cod specimens contained inflated STR abundances (up to 35%), which is far beyond the naturally observed levels (65). This inflation can be suppressed by a reduced number of amplification cycles and by the inclusion of synthesized dinucleotide repeat oligonucleotides during amplification. These data indicate that a biased amplification reaction, whereby repeats ‘*self-prime*’ during PCR, leads to artificially high levels of AC and AG repeats. Although this *self-priming* appears to be particularly problematic in cod—likely due to its high content of repeats with relatively low sequence complexity (65)—this process also explains the typical PCR fragmentation patterns observed when using transcript-activator like effector (TALE) technology (69). This highlights the propensity of repetitive DNA to interfere with amplification in a variety of protocols and conditions.

### Tandem-repeated gene families causing assembly collapse

Gene family expansions often originate from a gene locus being replicated in tandem, giving rise to two or more (almost) identical copies of a gene that can be regarded in essence as a long tandem repeat (70). Over time, these two copies can evolve independently, resulting in two genes with different function (neofunctionalization) or two genes with different expression patterns subfunctionalization). One such example is the α- and β-globin clusters in vertebrates, where multiple globin genes are found in tandem in each cluster, and where the different genes are expressed at different stages during the development (71). In teleost fishes, the two chromosomal regions are inhabited by different numbers of α− and β-genes, reflecting functional diversity (72). For instance, the different numbers of hemoglobin genes in codfishes are suggested to reflect the depth the different species are found at (i.e. a temperature-variation proxy) (73). Another gene family that greatly expanded in teleost fish are the nod-like receptor (NLR) genes (74,75), genes encoding proteins active in the innate immune system. It is not completely clear why this class of genes are expanded, but since they are involved in pathogen recognition the expansion might correspond to novel pathogen environments (75). In most teleost species, there does not seem to be a clear pattern to the genomic distribution of these genes (74), and although in many cases occurring as clustered (tandem) repeats they are also spread across the genome similar to transposable elements. Most notably, this multiplicity of similar sequences can cause local genome assembly collapse (i.e. the repeated genes are so similar that they collapse into one gene/region displaying much higher coverage than the rest of the genome) and annotation problems (i.e. annotated as a single gene while in reality multiple, or the genes might be hidden from annotation because the software register them as repeats). This problem can be illustrated by different releases of the zebrafish genome. In previous versions of this genome assembly (i.e. Zv6) the *NLR* genes were more or less collapsed. However, zebrafish assembly GRCz10 was created with substantial efforts in BAC and fosmid clones to close gaps, which enabled researchers to show that 159 of the 368 identified *NLR* genes are present as TRs on the long arm of chromosome 4 (76). As a further complicating repeat-issue they occur interspersed with Zn-finger genes and arranged irregularly. The specific organization of the *NLR* and *Zn-finger* genes is likely the result of multiple different local duplications. The repeated nature of this huge genomic architecture makes it difficult to be confident that all the genes have been properly assembled and annotated, even with manual annotation and curation (76).

Many immune genes such as NLRs contain leucine rich repeats (LRRs) (77). These are tandem repeats at the amino acid level, but not necessary at the nucleotide level. In jawless vertebrates the variable lymphocyte receptors (VLRs), another class of immune genes, also contain LRRs (78). In lamprey there are three *VLR* genes that each have multiple LRR-encoding modules in their vicinity. Together they can encode several hundreds of different proteins (78). During lymphocyte development, the *VLR* gene region is reorganised, ending up with the incorporation of several of the surrounding LRR modules. Different lymphocytes have different organisations of their *VLR* gene. In the sea lamprey assembly the *VLRC* gene is not complete and is found together with 182 different LRR donor genomic cassettes on 24 scaffolds (79). It is likely that the nature of these LRR cassettes make them hard to assemble properly, but this is not fully clear from the literature (79). An improved genome assembly of sea lamprey including PacBio reads has recently been published (80), but it remains to be seen if that assembly would resolve these complicated regions better.

**Figure 1.**
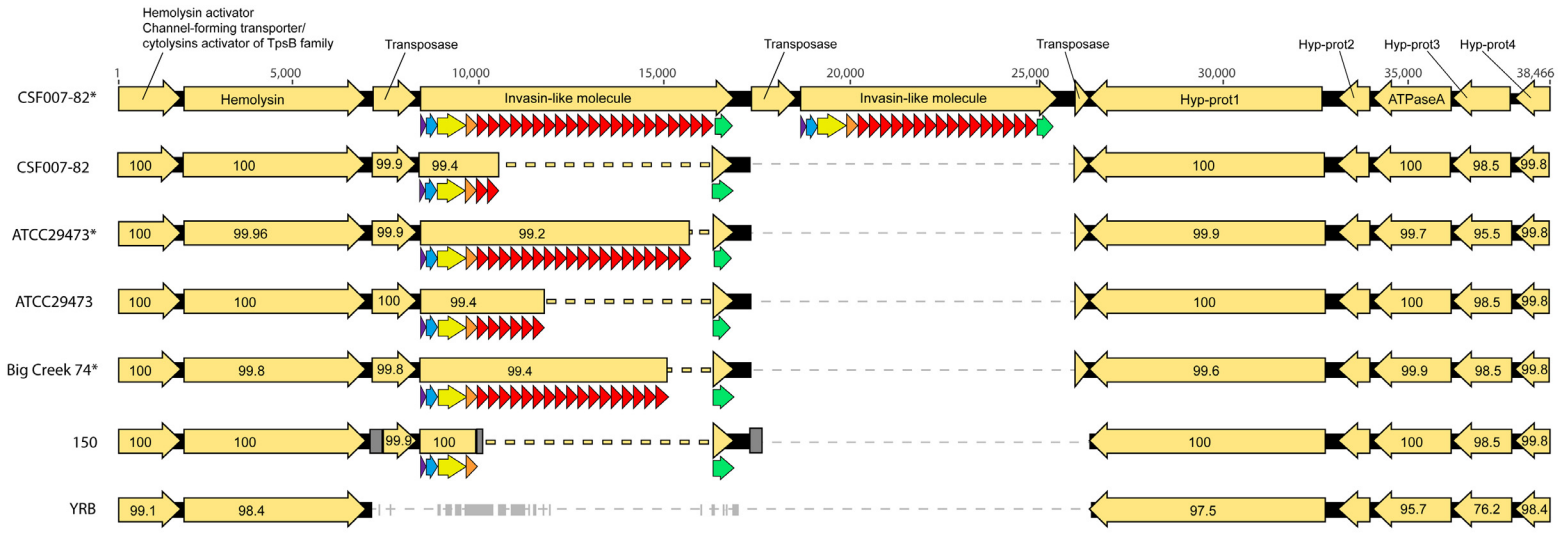
DNA alignment of a ∼39 kb-long DNA region containing the *yrIlm* gene and flanking CDS in *Y. ruckeri* genomes deposited in GenBank. Each CDS is indicated by a yellow arrow, with the percentage of sequence identity to CSF007-82 reported inside the arrow. *yrIlm* consists of an array of tandemly repeated, identical Ig-like domains (in red) and in addition of Ig-like domains of lower pairwise sequence similarity (in orange). It is usually capped by a C-type lectin domain (CTLD, in green). The dashed lines indicate gaps in the DNA alignment. In strain 150 the grey box indicates a contig break in the assembly. The asterisk (*) indicates assemblies generated through PacBio SMRT sequencing. Note that the other assemblies have significant lower repeat numbers, suggesting that the repeats were not found using short-read sequencing technologies. Modified from Wrobel,A., Ottoni,C., Leo,J.C., Gulla,S. and Linke,D. (2018) The repeat structure of two paralogous genes, Yersinia ruckeri invasin (yrInv) and a ‘Y. ruckeri invasin-like molecule’, (yrIlm) sheds light on the evolution of adhesive capacities of a fish pathogen. Journal of Structural Biology, 201, 171–183, with permission from Elsevier.

Long tandem repeats (LTRs) are often associated with protein-coding regions, and can include duplicated genes as well as duplicated (or otherwise multiplied) domains within a protein-coding gene. They are affected by the filtering and masking operations during genome assembly. A problem occurs when the read length of the sequencing method is shorter than the LTR—in this case, repeat numbers can be massively misjudged. In the case of protein-coding regions, this has direct effects on the interpretation of biological function. LTRs are not uncommon in structural proteins on cell surfaces, and in pathogenicity factors of bacteria, parasites, and viruses. As an example, Wrobel *et al.* (81) have shown that in the fish pathogen *Yersinia ruckeri*, a surface adhesin involved in biofilm formation called Ilm has >20 Ig-like domains repeated in tandem that are identical even on the DNA level (repeat length ∼300 bp). Repeat numbers vary slightly from strain to strain, but in this case only PacBio-based genomes show the correct number of repeats (Figure 1). Deposited genomes based on short-read methods show underestimated repeat numbers (by a factor of 4 to 5). The fact that the underestimated repeat number is an approximation made during genome assembly is not visible in the deposited genome data. In a very similar example, Franzén *et al.* find that in the human and animal parasite Giardia, variable surface proteins (VSPs) are difficult to sequence using 454 sequencing. Using this technology, only a few genes could be assembled due to their highly repetitive nature (82). From other experiments (including some re-sequencing using different technologies), the authors estimate that ca. 300 of these repetitive surface proteins should exist in the genome. In yeast, a large set of LTR proteins are included in flocculation (self-adhesion), a process important in biotechnology for removal of the yeast cells by sedimentation or filtration. These *flo* genes are often truncated in deposited genomes, but it is possible that in many cases, this is due to sequencing and assembly issues, and that in reality, these genes are intact in many of the sequenced strains (83). In primates, filaggrin protein is a component of the skin, and the underlying genes have copy number variations between different species (84). The gene contains multiple copies (10–12) of a repeat that is 972–975 nucleotides long. Here, researchers found incomplete versions of the gene for chimpanzee, gorilla, orangutan and macaque in the NCBI database, but were able to reconstruct the complete genes by using a combination of PacBio and Illumina sequencing (84), again showing the importance of the choice of sequencing technology. One extreme example of a LTR is *Pseudomonas koreensis* P19E3 where a 70 kbp repeat could not be resolved by PacBio sequencing reads (85). However, by utilizing very long reads from Oxford Nanopore in addition to PacBio and Illumina sequences, the researchers were able to properly resolve this LTR (85). Even in cases such as this, researchers may take different approaches to representing the sequence within the database. Guo *et al.* (86,87) identified a 37 kbp repeat in the *Marinomonas primoryensis* ice binding protein (MpIBP) but were unable to sequence through the region with PacBio sequencing. Based on pulsed-field gel electrophoresis they estimated that is contained about 120 copies of a 104 amino acid. When submitting the protein sequence, they deposited two sequences, one for the amino terminal side of the repeats and one for the carboxy terminal side of the repeats. In other cases such as the sequence determination of the R28 protein from *Streptococcus pyogenes* (88) the authors determined the sequence of the terminal repeats as well as random internal repeats derived from PCR and based on the estimated size of the PCR product of the complete repeat region deposited a full length sequence with an assumption that every repeat was identical.

It is worth noting that repeat numbers within coding regions may vary within a single bacterial colony, potentially leading to another level of complication when estimating repeat numbers. This effect is called hypervariable copy number variation; an example is the SasG protein from *Staphylococus aureus* strain NCTC 8325 which contains eight identical 128 amino acid B repeats. Roche and colleagues found that PCR of the full length SasG gene led to a ladder of products differing in size by the 400 bp repeat size (89). Individual bands were gel purified and used as a new template for PCR and in each case only a single band was identified demonstrating that the different size products were not due to mis-priming of the repeat DNA during amplification.

## ANNOTATION OF FUNCTION CAN BE AFFECTED BY TANDEM REPEATS

### Annotation of repeats

The task of accurate characterization of TRs should not rely on just one method. This is because the statistical error rates and power of TR prediction vary extensively for different repeat types and different methods - due to fundamental differences in prediction methodology and method assumptions (24). For example, the Tandem Repeats Finder program appears to be very conservative and has a very low power of predicting diverged repeats (Figure 3 in 24). As a result, the agreement of TR annotations by different methods is low, since different methods achieve optimal power for different subsets of TR space (in terms of TR unit length, repeat number and unit similarity). Indeed, testing four selected popular TR finders, Schaper and colleagues reported that 89% of TRs were found by only one program, <1% were found by three and only 0.2% by all four programs (24). To improve the accuracy and power of TR annotation, it is advisable to use a proper statistical framework combined with a meta-approach that employs several repeat prediction methods, followed by subsequent filtering of false positives using rigorous statistical tests (90). Currently, such procedure can be implemented using the Tandem Repeat Annotation Library (TRAL) (91). The TRAL library can be easily included in developing new pipelines for genome assembly and repeat annotation. Further, TRAL allows for evolutionary analyses of the annotated repeats, such as evaluating whether a TR region may be under selection.

A genome assembly is most useful when different features such as genes, TEs and other repeats are annotated with their precise location on a scaffold/chromosome and with a unique identifier. This can then provide essential background information for further experiments on gene expression or function, for example when investigating the difference in gene expression between two experimental set-ups with RNA-Seq (92). We often distinguish between structural annotation, specifying all the genes with their intron and exon structure, and functional annotation of genes and their properties (including individual function (e.g. for enzymes) or function in more complex pathways (e.g. in signaling)) (93,94). A key issue is the typical workflow of annotation in semi-automated pipelines. The annotation process starts with identifying as many repetitive elements as possible, possibly by creating a custom-made repeat library using both homology-based and *de novo* tools (95). Complete TEs often contain genes that are used to facilitate transposition and are often considered less important when investigating a particular species compared to the specific genes of that species. Repeat libraries are thus used to mask the repeats, making annotation of the genes of the species under investigation easier, but removing information related to genes found in transposable elements. TEs and TRs are usually masked. The reason for masking repeats is that *ab initio* gene prediction programs such as AUGUSTUS (96) or GeneMark (97) need to be trained, i.e., optimized for the specific species with regards to codon bias and splicing signals, and this training can be biased by repeats. Evidence for actively expressed genes can be added in the form of transcriptome data assembled by Trinity (98) or StringTie (99), or with the full-length transcripts generated by PacBio Iso-Seq (100). The transcriptome data is often crucial, since it - of the methods mentioned here - alone provide concrete evidence for the presence of the particular genes of a species, and not just assumed via prediction or mapping of proteins. Non-redundant protein databases such as UniProtKB/Swiss-Prot (101) can be included as the basis for annotation, ideally complemented by specific databases of well-annotated proteins from closely related species. All this information can then be integrated by using a program such as MAKER (102,103) or EVM (104). This approach provides a set of predicted transcripts and proteins, together with a GFF (General Feature Format) track with positions of all the annotated features, describing their properties. The predicted proteins can be searched using InterProScan (105) to classify proteins to different molecular functions, biological processes and pathways. Since such annotation is likely to be performed on assemblies where biologically relevant repetitive sequences have been removed from the data already, it may generate serious problems. The most important is the risk of removal of vital information about the genome from the final annotation. Consequently, if a TR makes up a large part of an exon or a whole gene, that exon or gene might not be properly annotated.

### Tandem repeats can hinder gene annotation

While the process above can already accidentally filter out genes with repetitive regions, the more detailed annotation process can add another level of problems. Specifically, homology search methods such as BLAST usually have built-in filters that hinder alignment to low complexity regions (which often exist as part of repetitive regions or are repetitive regions) (106), and are not adapted to accurately align homologous sequences with different numbers of TR units.

Therefore, the annotation process is often just a rough overview of the different genes, repeats and other features in the species of interest, and may not be sufficient for investigations into gene families that are particularly interesting for a researcher. Manual inspection, re-annotation and re-alignment are often necessary for troublesome gene families. One such gene family is the anti-freeze proteins, in particular the anti-freeze glycoproteins (AFGPs) of notothenioid fishes and codfishes (107,108). In nototheniods the AFGPs consist of a repeated pattern of Thr-Ala(/Pro)-Ala, and in codfishes it sometimes is represented by Arg-Ala(/Pro)-Ala (108). The repeated nature of these gene families requires manual annotation, and this was performed in a comparative survey of AFGPs in notothenioid fishes and codfishes (109). Indeed, the automated annotation of the Atlantic cod genome masked these genes as repeats and they would not have been properly characterized without careful investigation using BLAST (109). These genes were not properly assembled in the first version of the Atlantic cod genome (66), but were in the second version created with PacBio reads (62,109).

Detection of genuine gene fusion events has been reported long before the first complete genomes became available (110,111), but beyond that point they have been proven instrumental in detecting gene/protein associations with high specificity (112,113). Repeats may artificially cause gene fusion events, when genes/proteins that are encoded as distinct units in the genome under study (possibly in distant loci or even in different chromosomes). More specifically, in the case where the 5′ and 3′ termini of two gene loci share a similar repeat or low complexity pattern, there is an increased probability that genome assemblers can erroneously detect an overlap, thus artificially fusing these genes into a single entity. There are known cases where similar repeat regions in adjacent genes can lead to recombination-driven gene fusion (114), but with short sequence reads, assembly errors can arguably lead to ‘artificially’ fused genes (as detailed above). Such erroneous gene calls may (i) become the cause of downstream gene-prediction or annotation errors, (ii) generate false positive predictions for gene/protein associations and (iii) hinder large-scale genome evolution studies (115,116).

### Databases, submission and curation

DNA and protein sequences are routinely submitted to online repositories that make these data available to the public. This is a largely unsupervised process and there is usually little or no post-submission curation of the data. For nucleotide sequences, submitters must only ensure that the submission adheres to various formatting and data standards, and the archival database will make various automated checks of the data and metadata. Problems such as misassembly and contamination are not investigated. At the protein level, the UniProt database takes predicted sequences from nucleotide entries and places them within the UniProtKB/TrEMBL portion of the database with no further quality control. The RefSeq database, at least for bacterial genomes, ignores the submitted protein sequences and runs their own bespoke PGAP pipeline - this leads to a more consistent set of protein sequences and annotations. Only the manually reviewed section of UniProt, UniProtKB/Swiss-Prot allows for corrections to be made to protein sequences and curators will merge multiple entries from UniProtKB/TrEMBL, thus improving the likelihood of identifying the fully correct protein sequence. But even when manually curated, it is difficult to assess whether or not a protein contains the correct number of a repeated pattern or amino acid, and whether errors have occurred in the underlying DNA sequencing process. The difficulty of identifying and classifying DNA tandem repeats, in addition to their extreme variation from species to species, as well as within populations, has promoted the development of specialized bioinformatic algorithms and databases dedicated to repeat detection and characterization.

The first database on human repetitive DNA elements, including TRs, was developed in 1992 (117), eventually becoming RepBase (118). Widespread genome sequencing further fueled the development of specialized resources (both methods for detecting repeats and repeat databases). The parallel development of general and specialized resources related to DNA tandem repeats, has been crucial to the increased awareness of their widespread distribution and has been instrumental for their use both in basic and applied science. With over 50 TR detectors available, equally numerous repeat sequence databases exist today whose data is constantly used in practical applications like agriculture, medicine and forensics. Examples include the Human Genome Browser at UCSC (119), the STRBase (120) maintained by the National Institute of Standards and Technology (NIST, Maryland, US) or the Tandem Repeats Database (TRDB; (121)). Some of these databases have specific applications. For instance, the STRBase has a focus on human STRs whereas the TRDB was developed as a workbench for sequence analyses. Other specialized databases have been developed recently in this regard (e.g. (122–126)), starting off from human-centered research questions and expanding to examples of many other species, such as the tobacco plant (127), *Trichophytum rubrum*, a fungus causing skin disease (128), or the Cannabis plant to characterize the origin of hemp seeds (US Cannabis DNA database; (129)). Despite this diversity, the majority of these databases rely on the results of well-established automated bioinformatic approaches such as the Tandem Repeats Finder (TRF) program (130) or RepeatMasker (118) to characterize repeat content. Especially the use of RepeatMasker as *the* preferred software to identify and mask repeats, (http://www.repeatmasker.org/), has allowed the standardized treatment of raw genomic sequences and reproducibility of protocols for the establishment of these databases. However, using RepeatMasker and TRF on their own might not be enough to accurately characterize all TRs, and using a meta-approach such as TRAL (mentioned above) would likely lead to better annotation of TRs in both proteins and DNA.

## CONCLUSIONS

Both short and long repeat regions in genomes convey important biological functions; but as they cause significant technical problems with DNA sequencing, genome assembly, and gene and genome annotation, they often include significant errors, or are even omitted from datasets in public databases. Researchers with an interest in the function of such repeats may not be fully aware of the multi-level complexities and use genome data without questioning its quality. It is possible but not well documented that numerous publications on repeat numbers, gene duplications or recombination events are based on erroneous data and thus might include wrong evolutionary or functional conclusions. There is no easy solution to this issue and the key purpose of this article is to raise the awareness to the problem, especially amongst end-users of genome and protein databases, but likewise amongst the researchers working on sequencing, assembly and annotation projects that are often not fully aware of the biological importance of the repeat regions that they mis-sequence, mask, or remove. It would be beneficial if deposited data included qualitative and quantitative information on the type of sequencing methods used, the quality of the assembly and of the annotation. We strongly encourage the use of long-read sequencing technologies to better capture the tandem repeats at the sequencing and assembly stages. Specifically, we urge researchers to aim for a sequencing strategy similar to what has been decided for the Vertebrate Genome Project (not published, but partly described in (131) and on https://www.rockefeller.edu/research/vertebrate-genomes-project/technology-pipeline-and-policies/), and for Earth Biogenome Project (132). This sequencing strategy should in most cases lead to chromosome level genome assemblies for eukaryotes, where there are few gaps in the sequence and most repeats are resolved. For prokaryotes, substantial coverage in PacBio reads (60×), plus some Illumina reads (50×) and some coverage in very long Nanopore reads as described earlier would likely lead to complete prokaryote genome assemblies (85). It is important that more than one round of polishing with Illumina reads are performed on the assemblies, as that reduces any issues that might stem from the long reads (133,134). The combination of long and short reads has been shown to be beneficial for resolving tandem repeats in genomes (135), and it should create a better foundation for characterizing large gene families that might be underreported. Recent technological advances by PacBio have enabled circular consensus sequencing of both RNA and DNA, resulting in long (>10 kb), highly accurate (99.8%) reads (136). Wide-spread adoption of these technologies should address most of the issues raised here. While best-practice methods and quality control can improve new datasets that are made available to the research community, it is less clear how to manage the many problems found in existing, deposited data. More work should go into identifying such issues. It would be of great help if databases would allow user comments to deposited items, to alert other users of the problems and to avoid the reiteration of mistakes and misinterpretations. We expect that the wide-spread adaptation of such recommendations is improved by an increased awareness of the challenges associated with TRs within the community of database creators and end-users.

## Supplementary Material

gkz841_Supplemental_File

## APPENDIX

### Glossary

**aDNA:** Ancient DNA. DNA isolated from material that are up to several hundred thousand years old.

**Contigs:** Sequence assembled from shorter sequencing reads into a contiguous stretch of nucleotides.

**de Bruijn graph:** One of two main computational approaches (the other is **OLC**) for the assembly of sequencing reads into longer sequences such as contigs. Works by dividing reads into overlapping *k*-mers. A graph is created with nodes corresponding to *k*-mers and directional edges connecting overlapping nodes. A traversal of the graph can be output as contigs.

**GenBank:** One of several databases containing all publicly available DNA sequences.

**Homorepeat:** Also known as **homopolymer tract**, or **polyX** for amino acids, where X is the repeated residue. A perfect **tandem repeat** with unit size one where all the nucleotides or amino acids are the same.

**Interspersed repeat:** A motif or pattern that is found in multiple loci across a genome, such as **transposable elements**. In contrast, a **tandem repeat** has the motif or pattern repeated in tandem at one locus.

***K*-mer:** A sequence of nucleotides that is *k-*residues long, such as a 31-mer with 31 nucleotides.

**LRR: Leucine rich repeats** are amino acid motifs found in many different proteins, often repeated in tandem.

**NLR: Nod-like receptors** are proteins involved in innate immune response and contains LRRs among other domains.

**OLC: Overlap-layout-consensus**. One of two main computational approaches (the other is **de Bruijn graph**) for the assembly of sequencing reads into longer sequences such as contigs. Works by finding common sequences in reads (overlaps), and creates a graph where the overlaps are nodes. Traversal of the graph can be output as contigs.

**Polishing:** The act of mapping reads back to an assembly and recalling the consensus sequence. This is a necessity for assemblies based on PacBio and/or Oxford Nanopore reads, and are often performed in multiple rounds where at least the last couple are done with Illumina reads.

**Scaffolds:** Contains multiple contigs that are placed into proper order and orientation based on paired reads or other positional information (linked reads, optical maps, linkage maps).

**Short tandem repeat (STR):** A **tandem repeat** with a unit size shorter than 10 nucleotides.

**Sequence Read Archive (SRA):** A database of sequencing data and alignment information from high-throughput sequencing platforms such as Illumina, 454 and PacBio among others.

**Tandem repeat (TR):** A region of DNA or protein where a motif or pattern is repeated in tandem at one locus. The motif or pattern has a size, which is usually called a unit size. For example, the tandem repeat ACACACAC has a unit size of 2. This is in contrast to an **interspersed repeat** where the motif or pattern is found in multiple loci across a genome.

**Transposable elements (TE):** A class of repetitive elements that often code for their own propagation. Found across the genome as **interspersed repeats**.

**UniProtKB/Swiss-Prot:** A database of protein sequences that have been manually curated.

**VLRs: Variable lymphocyte receptors:** immune genes found in jawless vertebrates, also containing LRRs.
