# Supplementary material for: Tandem repeats lead to sequence assembly errors and impose multi-level challenges for genome and protein databases
Source: Nucleic Acids Res. 2019 Oct 4;47(21):10994–1006. doi: 10.1093/nar/gkz841 (PMC6868369; doi:10.1093/nar/gkz841)
Supplement: gkz841_Supplemental_File [file gkz841_supplemental_file.pdf]

Supplementary material

Number of entries of 454 sequences in SRA

In the NCBI SRA database (https://www.ncbi.nlm.nih.gov/sra/), a total of approximately $3*10^5$ entries are returned with the following query:
"ls454"[Platform]
Total number of SRA entries is approximately $6*10^5$. (query: ("pacbio smrt"[Platform] OR "bgiseq"[Platform] OR "capillary"[Platform] OR "complete genomics"[Platform] OR "helicos"[Platform] OR "illumina"[Platform] OR "ion torrent"[Platform] OR "ls454"[Platform] OR "oxford nanopore"[Platform] OR "pacbio smrt"[Platform])).

The vast majority are sequences derived from the Illumina platform (>$5*10^6$ entries), with the 454 platform being the second most numerous.

Sequencing errors in proteins

Methods

To analyse the number and length distribution of short tandem repeats we used two different approaches. In the first one, we checked how the number of repeats and their lengths changed in time when the new versions of sequences were submitted into UniProtKB/Swiss-Prot [1] database. In the second one, we checked if the distribution of the number of the same type of repeats in protein families can be questionably diverse. For both purposes, we analysed sequences from the UniProtKB/Swiss-Prot database using version 2018_06.
To identify and cluster repetitive regions we used our unpublished method (Jarnot, P., Ziemska-Legięcka, J., Grynberg, M., & Gruca, A., *in preparation*). It finds strings of repeats composed of one amino acid (homorepeats) or a few amino acids (STRs). This algorithm identifies and retrieves also imperfect tandem repeats from protein sequences by scanning all sequences in a database. Imperfect repeats mean that the algorithm allows for insertions in between repeats and mutations of amino acids within the repetitive region. The minimum length of homorepeats identified by the method is 6 and the minimum number of repeats in an STRs is 3. The position of a tandem repeat and the information about the type of repeat are collected for further analysis.

The clustering phase uses repeats and their assigned 'types' (classification) found during identification of tandem repeats. Additionally, one type of repeat can be followed by another type of repeat in the protein sequence, defined as 'fused repeats'. During clustering, fused repeats are also taken into account. Please note that if a protein contains three different STRs which are placed next to each other, then the method will produce 6 clusters: three with regular STRs and three with fused repeats.

In the first part of the analyses, we investigated lengths of repetitive regions between different versions of the same protein sequences (available at uniprot.org). For each sequence from the UniProtKB/Swiss-Prot we retrieved the latest (version 2018_06) and the first version. We aligned the sequences using KAlign [2] with default parameters and trimmed non-repeated parts of the sequences where the two versions differed (for example overhangs). This left common parts and STRs (Figure 1). We then retrieved STRs from these sequences to finally analyse the difference in length of these repetitive regions.

```
MESQQDEAVQTKGASTSSDAQDQGAEKGAKNKTTEATEGPTSEPPLSGPGRLKKTAMKLF
MESQQDEAVQTKGASTSSDAQDQGAEKGAKNKTTEATEGPTSEPPLSGPGRLKKTAMKLF

GGKKGICTLPSFFGGGRSKGSGKVSSKKSLNKSKTHDGLSEASQGPEDVVIEETDLSTPL
GGKKGICTLPSFFGGGRSKGSGKVSSKKSLNKSKTHDGLSEASQGPEDVVIEETDLSTPL

SKSSAQFPSSQSANGALEIGSKHKTSGTEAIEKAGVEKVPSVHKPKKSLKSFFSSIRRHR
SKSSAQFPSSQSANGALEIGSKHKTSGTEAIEKAGVEKVPSVHKPKKSLKSFFSSIRRHR

KGKTSGADQSVPGAKELEGARTRSHEHVSSISLPSSEEIFRDTRKENAKPQDAPGPKMSP
KGKTSGADQSVPGAKELEGARTRSHEHVSSISLPSSEEIFRDTRKENAKPQDAPGPKMSP

AQVHFSPTTEKAACKNPEKLTRTCASEFMQPKPVLEGGSLEEPHTSETEGKVVAGEVNPP
AQVHFSPTTEKAACKNPEKLTRTCASEFMQPKPVLEGGSLEEPHTSETEGKVVAGEVNPP

NGPVGDQLSLLFGDVTSLKSFDSLTGCGDIIAEQDMDSMTDSMASGGQRANRDGTKRSSC
NGPVGDQLSLLFGDVTSLKSFDSLTGCGDIIAEQDMDSMTDSMASGGQRANRDGTKRSSC

LVTYQGGGEEMALPDDDDNDDEEEEEEEEKKKKKKKKKKKKKKKK---------------
LVTYQGGGEEMALPDDDDNDDEEEEEEEEEEEEEEEEEEEEEEEEEEEELLEDEEEVKDG

------------------------------------------------------------
EENDDLEYLWASAQIYPRFNMNLGYHTAISPSHQGYMLLDPVQSYPNLGLGELLTPQSDQ

------------------------------------------------------------
QESAPNSDEGYYDSTTPGFEDDSGEALGLAHRDCLPRDSYSGDALYEFYEPDDSLEHSPP

------------------------------------------------------------
GDDCLYDLRGRNSEMLDPFLNLEPFSSRPPGAMETEEERLVTIQKQLLYWELRREQREAQ
```

```
------------------------------------------------------------
EACAREAHAREAYARDTHTRESYGRNVRARETQALEAHSQEGRVQETKVRQEKPALEYQM

------------------------------------------------------------
RPLGPSVMGLVAGTSGGSQTSHRGTTSAFPATSSSEPDWRDFRPLEKRFEGTCSKKDQST

------------------------------------------------------------
CLMQLFQSDAMFEPDMQEANFGGSPRKAYPSYSPPEEPEEEEEEKEGNATVSFSQALVEF

------------------------------------------------------------
TSNGNLFTSMSYSSDSDSSFTQNLPELPPMVTFDIADVERDGEGKCEENPEFNNDEDLTA

------------------------------------------------------------
SLEAFELGYYHKHAFNSYHSRFYQGLPWGVSSLPRYLGLPGVHPRPPPAAMALNRRSRSL

------------------------------------------------------------
DNAESLELELSSSHLAQGYMESDELQAHQEDSDEEGEEEEGEWGRDSPLSLYTEPPGVYD

------------------------------------------------------------
WPPWAHCPLPVGPGLAWMSPNQLYEPFNQSSYVQATCCVPPVAMPVSVPGRTPGDSVSQL

------------------------------------------------------------
ARPSHLPLPMGPCYNLQSQASQSGRAKPRDVLLPVDEPSCSSISGANSQSQAKPVGITHG

----------------------------------------------------
IPQLPRVRPEPFQLQPNHYRASNLDLSKERGEQGASLSTSYSSTAMNGNLAK
```

Supplementary Figure 1. Identification of repeats differences in sequences of APC membrane recruitment protein 1, *Mus musculus (Mouse)* (Q7TS75). The blue part is common to both sequences and this part is analysed. Red part is omitted.

The second part of our analyses focused on describing the differences in STRs length in specific protein families. For that purpose, we divided the UniProtKB/Swiss-Prot database into following taxonomies: Archaea, bacteria, fungi, invertebrates, vertebrates, plants and viruses. In the next step, we retrieved STRs from each sub-database and clustered them by type of repeats and families. Then we generated statistics for each cluster in order to find differences in lengths in repetitive regions in the same families for particular taxonomies.

Results

We found that in the UniProtKB/Swiss-Prot database 1669 (0.3%) proteins have differences in repetitive regions between the first and the last (current) submitted version of the protein sequence. These regions vary in length and quantity of repeats. The average absolute difference is 13.57 amino acids. The average length of the repetitive region in the first version of sequences is 31.14 whereas in the current version it is 35.2. The results of our analysis are summarised in Table 1.

While analysing the distribution of STRs in protein families for specific taxonomies we found out that 12.21% of invertebrate proteins contain short tandem repeats, especially PolyQ and PolyN, and many of them are characterized by a large variation in length within the same family. For instance the paralogs of probable serine/threonine-protein kinase dyrk1 (Q76NV1, Q54V83, *Dictyostelium discoideum*) are quite similar in case of high complexity regions, however PolyN repetitive regions in the first protein which is positioned in range 107-276 is almost 4 times longer and more regular, i.e., fewer insertions and mutations, than the corresponding region in the second protein (10-53).

Another example is the pair: probable basic-leucine zipper transcription factor O (Q54GH0, *Dictyostelium discoideum*) and the CCAAT/enhancer-binding (Q02638, *Drosophila virilis*) proteins. If we align both sequences using MUSCLE tool [2], it reveals that PolyQ region (28-76) is over twice longer in the first protein than in the second protein (47-69).

We discovered that another group of organisms that are also abundant in STRs are fungi. 11.89% of fungi proteins contain STRs. In contrast to invertebrates, differentiation in fungi is more visible in non-hompolymeric repeats. For instance the protein sequences of DNA-directed RNA polymerase II subunit rpb1 from *Schizosaccharomyces pombe* (P36594) and RNA polymerase II subunit rpb1 from *Encephalitozoon cuniculi* (Q8SSC4) possess recurring regions consisting of the YSPTSPSYS subsequence at the C-terminus. This region occurs in the ranges 1553-1752 and 1466-1572, respectively, therefore the STR in the S. pombe sequence is almost twice as long as in its *E. cuniculi* counterpart. Significant difference in length can also be observed in proteins described as Mediator of RNA polymerase II transcription subunit 15 (Q75BI6 and Q9Y808) from *Ashbya gossypii* and *S. pombe*, which have glutamine homorepeats at ranges 282-365 and 256-289, respectively. Therefore, this first STR is 252% longer than the same region in the second protein.

Short tandem repeats in vertebrates are even more complex than in fungi, even if only about 8% of proteins contain STRs. Histone-lysine N-methyltransferase 2D (Q6PDK2, *Mus musculus*) which was added to UniProt database November 30, 2010 contains significantly more homorepeats of glutamine than histone-lysine N-methyltransferase 2C (Q8BRH4, *Mus musculus*) which was added to the database in October 10, 2003. Overall, there seems to be more STRs in more recent SwissProt additions.

Length variation of STRs in Archaea is very low. That is because proteins of these organisms are rarely composed of STRs. About 1.8% of proteins contain STRs. STRs in Archea proteins are mostly composed of A, E, Q, G, K amino acids.

Summary

In this research, we have shown that with new methods of sequencing, the number of repetitive regions in proteins changed significantly as well as the length of these regions.

Proteins in the same families share similar biological function. It has been shown that repetitive regions can have crucial functions in proteins [3]. These functions are related to the length of repetitive regions, therefore if a specific repetitive region has an important function in a protein, then the length of this repetitive region should not vary a lot within the protein family. Here we have shown some cases where the length of repetitive regions in the same family varies significantly.

Conclusion

By analysis of the different versions of the same protein sequences submitted to UniProtKB/Swiss-Prot database, we have shown that with the improvement of sequencing methods numbers of repeats and their lengths may change significantly. Additionally, we analysed the differences between the distribution of STRs length in specific protein families for particular taxonomies. Our results show that repetitive regions in the same taxon and family may vary significantly. These statements lead us straight to hypothesise that there are still many repetitive regions in UniProtKB/Swiss-Prot database which are erroneously sequenced.

References

1. The UniProt Consortium. UniProt: the universal protein knowledgebase. Nucleic Acids Res. 45: D158-D169 (2017)
2. Timo Lassmann and Erik L.L. Sonnhammer (2005). Kalign - an accurate and fast multiple sequence alignment algorithm. BMC Bioinformatics 6:298
3. Edgar, R.C. (2004) MUSCLE: multiple sequence alignment with high accuracy and high throughput. Nucleic Acids Res. 32(5):1792-1797.
4. Hamm D.C., Bondra E.R., Harrison M.M. Transcriptional activation is a conserved feature of the early embryonic factor zelda that requires a cluster of four zinc fingers for DNA binding and a low-complexity activation domain. The Journal of Biological Chemistry. 2015;290(6):3508-3518.
